# Radiation-triggered psoriasiform rash with systemic dissemination after prolonged PD-1 inhibitor therapy: A case report

**DOI:** 10.1097/MD.0000000000045550

**Published:** 2025-11-07

**Authors:** Mingjun Wu, Qian Yang, Yan Hou, Haizhen He, Youcheng Xie, Qingliang Xue

**Affiliations:** a Respiratory, 940th Hospital of Joint Logistic Support Force of Chinese People’s Liberation Army, Lanzhou, Gansu Province, China; b Department of Plateau Medicine, 940th Hospital of Joint Logistic Support Force of Chinese People’s Liberation Army, Lanzhou, Gansu Province, China; c Central Sterile Supply Department, 940th Hospital of Joint Logistic Support Force of Chinese People’s Liberation Army, Lanzhou, Gansu Province, China.

**Keywords:** abscopal effect, immune-related adverse event, PD-1 inhibitor, psoriasiform rash, radiotherapy, tislelizumab

## Abstract

**Rationale:**

Psoriasiform rash associated with programmed cell death protein 1 (PD-1) inhibitors typically occurs during the early phase of treatment. However, systemic dissemination triggered by radiotherapy after more than 2 years of immunotherapy is rarely reported. This case aims to highlight the potential for delayed and severe cutaneous immune-related adverse events following combined immunotherapy and radiotherapy, which has significant implications for long-term patient monitoring. Herein, we present a case of a patient with driver-negative lung adenocarcinoma who, after 25 months of tislelizumab monotherapy without cutaneous toxicity, subsequently developed a unique clinical course of psoriasiform rash. The rash initially emerged at the irradiation site (right iliac crest) 1 month after local radiotherapy and progressively disseminated systemically.

**Patient concerns:**

An elderly patient with lung adenocarcinoma, having received tislelizumab for 2 years, developed a right iliac bone metastasis and subsequently underwent stereotactic body radiotherapy (45 Gy in 5 fractions), achieving complete pain relief. However, 1 month post-radiotherapy (25 months after initiating immunotherapy), well-demarcated scaly plaques initially appeared at the irradiation site, followed by centrifugal dissemination to the scalp, trunk, and limbs.

**Diagnoses:**

Psoriasis as a cutaneous immune-related adverse event.

**Interventions:**

PD-1 inhibitor therapy was discontinued, and treatment with oral prednisone (0.5 mg/kg/day) combined with topical halometasone was initiated.

**Outcomes:**

After 4 weeks of treatment with oral prednisone (0.5 mg/kg/day) and topical halometasone, the psoriasiform rash showed marked regression, with > 80% reduction in erythema and scaling. The patient reported significant relief from pruritus and no new lesions emerged. PD-1 inhibitor therapy remained discontinued, and the patient continued under dermatological surveillance.

**Lessons:**

This case supports the “two-hit hypothesis”: prolonged PD-1 inhibition establishes a subclinical autoimmune state (first hit), and radiotherapy acts as a second hit, ultimately culminating in systemic toxicity. These findings underscore the necessity for long-term cutaneous surveillance during immunotherapy and rigorous dermatological assessment when combined with radiotherapy.

## 1. Introduction

Cutaneous toxicity is a common immune-related adverse event (irAE) associated with programmed cell death protein 1 (PD-1) inhibitors, occurring in 30% to 40% of patients, with psoriasiform rash gaining increasing attention as a distinct subtype.^[1]^ Current clinical observations indicate that PD-1 inhibitor-induced psoriasis primarily manifests during the early phase of treatment (<6 months).^[2]^ Furthermore, radiotherapy (RT) can enhance the antitumor efficacy of PD-1 inhibitors via the “abscopal effect,” but this synergy may also exacerbate immune toxicity.^[3]^ Here, we report the first case of systemic psoriasiform rash triggered by local radiotherapy after prolonged (>2 years) maintenance therapy with tislelizumab. Through systematic analysis of its clinical progression and pathological mechanisms, this case provides novel evidence for the management of long-term immunotherapy toxicity.

With the widespread application of immune checkpoint inhibitors in various advanced malignancies, their systemic treatment strategies continue to evolve, significantly improving patient survival outcomes.^[4]^ However, the patterns of response to immunotherapy and the occurrence of irAEs exhibit considerable interindividual heterogeneity. Predictive factors, including tumor microenvironment characteristics, host immune status, and treatment strategy selection, remain key focuses and challenges in current clinical research.^[5–7]^ Particularly in the context of increasingly prevalent combination immunotherapies, identifying complex or uniquely presenting irAE patterns poses new challenges for patient safety management.^[8,9]^ Against this backdrop, in-depth exploration of delayed-onset, radiotherapy-induced, or unusually presenting irAEs is not only crucial for optimizing patient management strategies but also holds significant scientific value for elucidating the mechanisms regulating immune homeostasis.

## 2. Case report

**Figure 1.**
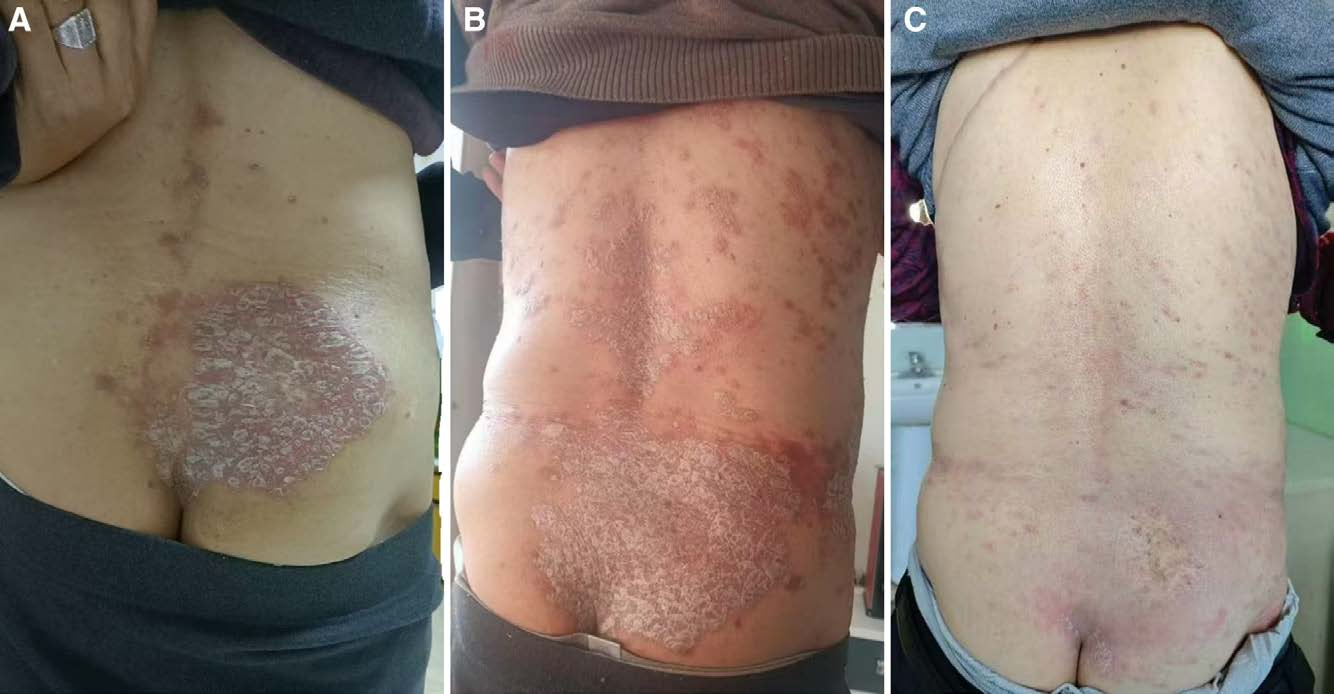
(A) A well-defined psoriasiform rash appears in the right iliac crest region. (B) The psoriasiform rash has spread to the entire body. (C) The rash shows significant regression after treatment.

**Figure 2.**
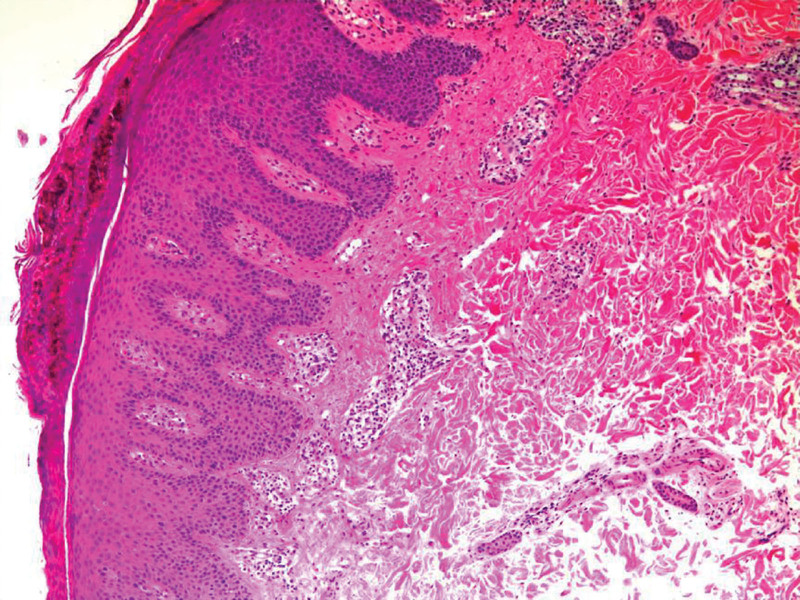
Skin biopsy shows hyperkeratosis with parakeratosis, elongated rete ridges, and neutrophilic microabscesses around superficial dermal vessels.

An elderly patient underwent radical left upper lobectomy in June 2022 for a lung mass detected on CT, with postoperative pathology confirming poorly differentiated lung adenocarcinoma (pT2aN0M0, driver gene-negative). Adjuvant therapy consisted of 6 cycles of nab-paclitaxel (400 mg D1), carboplatin (AUC 5), and bevacizumab (400 mg). In January 2023, progressive abdominal pain developed, and PET-CT confirmed multiregional lymph node metastases (mediastinal, abdominal, and retroperitoneal). Following multidisciplinary discussion, second-line tislelizumab monotherapy (200 mg q3w) was initiated, achieving partial response with sustained tumor regression. In January 2025, the patient developed right iliac bone metastasis (with osteolytic destruction confirmed by CT) and underwent stereotactic radiotherapy (45 Gy/5 fractions), resulting in complete pain relief. However, 1 month post-radiotherapy (after 25 months of immunotherapy), well-demarcated scaly plaques first appeared at the irradiation site (Fig. 1A), followed by centrifugal dissemination to the scalp, trunk, and extremities (Fig. 1B). Skin biopsy revealed hyperkeratosis with parakeratosis, elongated rete ridges, and neutrophilic microabscesses in the superficial dermis (Fig. 2), consistent with psoriatic pathology. Graded as G3 cutaneous irAE per ASCO criteria, PD-1 inhibitor therapy was discontinued, and prednisone (0.5 mg/kg/day) combined with topical halometasone was initiated. After 4 weeks of treatment with oral prednisone (0.5 mg/kg/day) and topical halometasone, the psoriasiform rash showed marked regression, with > 80% reduction in erythema and scaling(see Table 1). The patient reported significant relief from pruritus and no new lesions emerged. PD-1 inhibitor therapy remained discontinued, and the patient continued under dermatological surveillance (Fig. 1C).

**Table 1:** Clinical timeline of diagnosis, treatment, and adverse event.

Timepoint	Significant event
June 2022	▪ Diagnosis of lung adenocarcinoma (pT2aN0M0) following left upper lobectomy.
July 2022–December 2022	▪ Completion of 6 cycles of adjuvant chemotherapy.
January 2023	▪ Detection of metastatic disease in mediastinal, abdominal, and retroperitoneal lymph nodes. ▪ Initiation of tislelizumab (200 mg q3w).
January 2023–December 2024	▪ Maintenance tislelizumab. ▪ Partial response (PR) achieved. ▪ No significant cutaneous toxicity reported throughout this period.
January 2025	▪ Diagnosis of right iliac bone metastasis. ▪ Administration of stereotactic radiotherapy (45 Gy/5 fractions).
February 2025 (1 month post-RT)	▪ Initial emergence of psoriasiform rash at the irradiation site. ▪ Subsequent centrifugal dissemination to the scalp, trunk, and extremities.
February 2025 (post-diagnosis)	▪ Tislelizumab discontinued. ▪ Initiation of prednisone (0.5 mg/kg/day) and topical halometasone.
March 2025 (4 weeks post-treatment)	▪ Significant regression of psoriasiform rash observed.

There was no history of disease. There was no history of surgery or trauma. There was no history of food or drug allergy, and no relevant personal or family history.

## 3. Discussion

The pathophysiology of PD-1 inhibitor-induced psoriasis involves multifaceted immune dysregulation. The PD-1/PD-L1 axis plays a critical role in maintaining peripheral immune tolerance, and its inhibition disrupts immune homeostasis. Studies indicate that PD-1 signaling finely modulates the Th17/Treg balance via the mTOR pathway. Inhibiting this axis impairs Treg function while aberrantly activating Th17 cells, leading to excessive release of pro-inflammatory cytokines (e.g., IL-17A, IL-22) and subsequent keratinocyte hyperproliferation.^[1]^ Notably, in genetically predisposed individuals (e.g., carriers of the HLA-C*06:02 allele), PD-1 inhibitors may reactivate autoreactive T-cell clones targeting cutaneous antigens (such as keratin or LL-37).^[10,11]^ Emerging evidence also highlights the epigenetic impact of PD-1 blockade, particularly DNA methylation changes in CD8 + T cells that enhance tissue infiltration,^[12]^ offering a novel molecular perspective on cutaneous irAEs.

The synergistic triggering mechanism of radiotherapy in this case warrants in-depth analysis. Despite 25 months of immunotherapy without cutaneous toxicity, the localized rash emerged within 1 month post-radiotherapy and rapidly progressed to systemic involvement. This temporal pattern underscores the role of radiotherapy in disrupting immune equilibrium through multiple pathways. Ionizing radiation induces immunogenic cell death in keratinocytes, releasing damage-associated molecular patterns and autoantigens.^[13,14]^ Furthermore, radiotherapy upregulates endothelial adhesion molecules (e.g., ICAM-1, VCAM-1), facilitating inflammatory cell infiltration. The Koebner phenomenon may further contribute to the development of psoriasiform lesions in genetically susceptible individuals.

This case provides crucial insights into the management of long-term irAEs. Even after 2 years of immunotherapy, radiotherapy can still precipitate a delayed-onset irAE, emphasizing the pivotal role of local tissue injury in triggering systemic autoimmunity. Encouragingly, glucocorticoids demonstrated significant efficacy, offering a valuable reference for the management of similar cases.

This case represents the first reported instance of systemic psoriasiform rash triggered by radiotherapy after prolonged tislelizumab therapy. Its unique clinical pattern of “long latency - local initiation - systemic dissemination” provides strong clinical evidence for the “two-hit hypothesis” of immune checkpoint inhibitor-related toxicity. This finding alerts clinicians that even after over 2 years of immune monotherapy without significant toxicity, the potential for severe delayed-onset irAE following combined local radiotherapy cannot be overlooked. In clinical practice, sustained vigilance, continuous cutaneous monitoring, and follow-up are imperative for patients receiving combined immunotherapy and radiotherapy. Furthermore, this case highlights the critical role of local tissue injury events (e.g., radiotherapy) in breaking immune balance and triggering systemic autoimmune reactions, offering important clinical implications for further research into the pathogenesis of irAEs.

However, significant knowledge gaps in this field remain to be addressed. Firstly, there is a critical lack of biomarkers capable of predicting such delayed-onset, radiotherapy-triggered irAEs. Future studies could dynamically monitor cytokine profiles (e.g., IL-17, IL-6, TNF-α levels), autoantibody titers, and the evolution of specific T-cell clones in patients before and after radiotherapy to identify early predictive indicators. Secondly, the immunological characteristics of the “subclinical autoimmune state” induced by immunotherapy are poorly understood. Utilizing preclinical models and applying high-dimensional immune profiling techniques (e.g., single-cell RNA sequencing, T-cell receptor sequencing) to serially analyze patient biospecimens will help elucidate the cellular and molecular basis of this state. Finally, the optimal clinical management strategy for these rare but severe irAEs remains undefined. Beyond systemic glucocorticoids, should biologic agents targeting specific inflammatory pathways (e.g., the IL-17/IL-23 axis) be introduced early? What are their efficacy and safety profiles? These questions urgently require answers through the collection of more similar case data and the conduct of prospective studies.

Looking ahead to the next 5 years, with the continuous expansion of immunotherapy indications, prolonged treatment durations, and the increasing adoption of combinations with local therapies like radiotherapy, we anticipate that more delayed-onset or unusual types of irAEs will be identified and reported. Integrating multi-center data and real-world evidence holds promise for building more precise irAE risk prediction models incorporating multidimensional factors such as tumor type, immunotherapy agent and duration, radiotherapy parameters, and host genetic background. Ultimately, we hope to witness a shift in immune toxicity management strategies from reactive “post-occurrence treatment” to proactive “prediction and prevention,” leveraging mechanism-based targeted interventions to maximize antitumor efficacy while enhancing long-term treatment safety for patients.

## 4. Limitations

This study is limited by its nature as a single-case report, which restricts the generalizability of the findings. The mechanisms underlying the delayed irAE remain speculative, and the lack of serial immune profiling or cytokine monitoring precludes a deeper mechanistic understanding. Future multi-center studies with larger cohorts are needed to validate these observations and identify predictive biomarkers.

## 5. Conclusion

We report the first documented case of systemic psoriasiform rash triggered by radiotherapy after prolonged tislelizumab therapy. Its unique pattern of “long latency - local initiation - systemic dissemination” enhances our understanding of the spatiotemporal dynamics of immunotherapy toxicity. Our findings support the “two-hit hypothesis”: prolonged PD-1 inhibition induces a subclinical autoimmune state, and radiotherapy serves as the second hit, ultimately leading to systemic toxicity. This underscores the necessity for long-term cutaneous surveillance during immunotherapy and rigorous dermatological assessment when combined with radiotherapy. Future research should investigate the dynamic changes of key cytokines (e.g., IL-17), develop predictive biomarkers, and formulate targeted preventive strategies to optimize the safety of combined immunotherapy and radiotherapy.

## Author contributions

**Conceptualization:** Mingjun Wu, Qian Yang.

**Funding acquisition:** Youcheng Xie.

**Resources:** Haizhen He.

**Writing – original draft:** Mingjun Wu, Qian Yang.

**Writing – review & editing:** Yan Hou, Qingliang Xue.
